# Study Protocol for the Evaluation of Multidisciplinary Medication Reconciliation Service in Adult Patients Undergoing Thoracic and Cardiovascular Surgery (The MERITS Study): A Single-Center Controlled before-and-after Study

**DOI:** 10.3390/healthcare11121778

**Published:** 2023-06-16

**Authors:** Soyoung Park, A Jeong Kim, Hyun-Woo Chae, Kyu-Nam Heo, Yookyung Kim, Sung Hwan Kim, Yoon Sook Cho, Hyun Joo Lee, Ju-Yeun Lee

**Affiliations:** 1 College of Pharmacy and Research Institute of Pharmaceutical Sciences, Seoul National University, Seoul 08826, Republic of Korea; sesthdud2003@snu.ac.kr (S.P.); hwchae@snu.ac.kr (H.-W.C.); bogopa8@snu.ac.kr (K.-N.H.); 2 Department of Pharmacy, Seoul National University Hospital, Seoul 03080, Republic of Korea; anemone@snuh.org (A.J.K.); 30647@snuh.org (Y.K.); 30349@snuh.org (S.H.K.); 30108@snuh.org (Y.S.C.); 3 Department of Thoracic and Cardiovascular Surgery, Seoul National University Hospital, Seoul National University College of Medicine, Seoul 03080, Republic of Korea

**Keywords:** medication reconciliation, multidisciplinary, transition of care, best possible medication history

## Abstract

Medication reconciliation (MR), which is widely implemented worldwide, aims to improve patient safety to reduce the medication errors during care transition. Despite its widespread use, MR has not yet been implemented in the Republic of Korea, and its effectiveness has not been studied. We aimed to evaluate the impact of a multidisciplinary MR service in older patients undergoing thoracic and cardiovascular surgery. This is a single-center, prospective, controlled, before-and-after study of adult patients taking at least one chronic oral medication. Depending on the period of each patient’s participation, they will be allocated to an intervention group or control group. Patients in the intervention group will receive multidisciplinary MR, and those in the control group will receive usual care. The primary outcome is to assess the impact of the MR service on medication discrepancies between the best possible medication history and medication orders at care transition. Secondary outcomes include the incidence rate of medication discrepancies at each transition, the discrepancy rate between the sources of information, the impact of MR on medication appropriateness index score, drug-related problems, 30-day mortality, the emergency department visit rate, readmission rate after discharge, the rate and acceptability of pharmacists’ intervention during hospitalization, and patients’ satisfaction.

## 1. Introduction

Transition of care can be a vulnerable moment for patients, as it often involves changing medication regimen and medication discrepancies, and gaps in communications can lead to medication errors and potential harm. According to a systematic review, 27%–54% of patients had at least one unintentional medication discrepancy that could result in drug-related problems (DRPs), which actually or potentially harmed patients [1]. Ema et al. found that 63.7% of patients experienced at least one DRP after the discharge and also showed that DRPs were more likely to occur when patients had multiple medication changes [2]. Moreover, among the medication errors related to reconciliation issues, 22% occurred during patient admission, 12% occurred during discharge and one-third occurred during the transition of care [3].

To improve medication safety at transition points of care, medication reconciliation (MR) was first adopted as a National Patient Safety Goal by the Joint Commission in 2005 and suggested as an imperative process by international health organizations [4]. In addition, the World Health Organization (WHO) has prioritized this area as a critical commitment to protect patients from medication-related harm during transitions of care [5].

MR is defined as a formal process of identifying an accurate list of a person’s best possible medication history (BPMH) and comparing it with the current list in use, recognizing any discrepancies and documenting any changes. The BPMH is obtained through a systematic process that involves interviewing the patient, their family members or caregivers. The information gathered during the interview is then verified by cross-referencing it with at least one other reliable source of information [4]. Previous systematic reviews have shown that MR significantly reduces the rate of adverse drug events that lead to outpatient visits by 67%, emergency department visits by 28%, and hospital readmissions by 19% compared to usual care [6]. Furthermore, a systematic review published in 2019 reported that the risk of medication errors was 75% lower when MR was conducted (risk ratio 0.25; 95% CI 0.15–0.43) [7].

While MR has been implemented in routine clinical practice in many countries worldwide [5], the systematic introduction and implementation of MR services have not yet been established in the Republic of Korea. The lack of understanding or awareness regarding the incidence of medication discrepancies or DRPs during care transition periods in the Republic of Korea may hinder the active adoption of this system.

We will attempt to introduce and evaluate the “Multidisciplinary Medication Reconciliation service in adult patients undergoing Thoracic and cardiovascular Surgery” (MERITS trial). The primary goal is to assess the impact of the multidisciplinary MR service on medication discrepancies during transitions of care. By evaluating these outcomes, the MERITS trial hopes to demonstrate the benefits of a multidisciplinary MR service in improving medication management and patient outcomes in transitional care.

## 2. Materials and Methods

### 2.1. Design

This study was reviewed and approved by the Institutional Review Board of Seoul National University Hospital (IRB No 2109-135-1257). This is a single-center, prospective, controlled, before-and-after study comparing two patient care strategies: (1) the control group, receiving standard care; (2) the intervention group, receiving multidisciplinary MR service. Depending on the time period of each patient’s participation, they will be assigned to either the control group or the intervention group. The study will initially enroll patients into the control group. Once the enrollment for the control group is completed, the study will proceed to enroll patients into the intervention group. Study enrolment started in December 2021, and the study is expected to be completed within 3 years. There are two phases in this study. Phase 1 (control group) will last about four months, and phase 2 (intervention group) will last eight months.

### 2.2. Setting

This study will be carried out in the Seoul National University Hospital (SNUH). It is located in the metropolitan area, and is a 1793-bedded tertiary hospital. The study will be conducted in five wards where patients undergoing thoracic and cardiovascular surgery are hospitalized.

### 2.3. Inclusion and Exclusion Criteria

**Figure 1:**
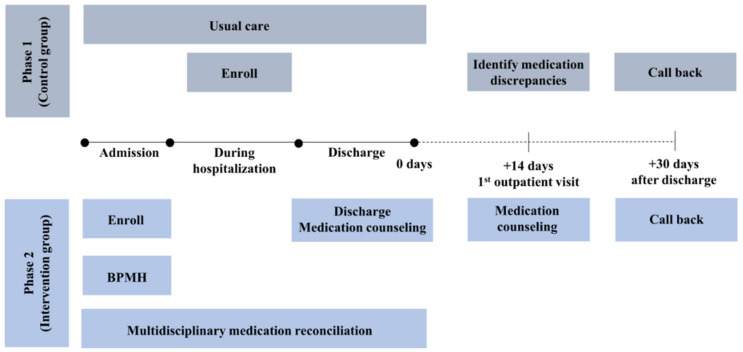
Flow of the intervention group (Phase 2) and control group (Phase 1).

Patients undergoing thoracic and cardiovascular surgery during the study period will be eligible for inclusion if they are 65 or older, are vulnerable to DRP [8,9] and are taking more than one chronic oral medication for an underlying comorbid disease before surgery. Patients who (1) have less than 48 h of hospital stay, (2) are unable to understand or complete questionnaires, (3) died during hospitalization, (4) are lost to follow-up, or (5) are considered inappropriate to enroll, for example, for being unable to understand Korean or having a diagnosis of dementia, by the medical staff will be excluded. Patients who meet all inclusion criteria and none of the exclusion criteria will be asked to participate in the study and provided with all the relevant information both verbally and in writing. The patients will not be informed about which group they have been assigned to in order to maintain blinding. If they decide to participate, they will be asked to sign an informed consent form and assigned to a group (Figure 1).

### 2.4. Intervention

#### 2.4.1. Phase 1: Control group

Patients enrolled in the control group will receive the usual standard of care. Pre-admission medications will be obtained by nurses. During hospitalization, health professionals will measure frailty according to the Korean-translated version of the Clinical Frailty Scale (CFS) [10]. Patients will receive discharge medication counseling as a part of discharge summary from the medical team. However, pharmacists will not be involved in the discharge process. At the first outpatient visit after the discharge, which will take place around 14 days after the discharge, the patients in the control group will receive face-to-face medication counseling from pharmacists. The counseling will include an assessment of the patients’ preadmission medications, and an identification of any discrepancies and DRPs. The patients will also be asked whether they had any ED visits or unscheduled readmissions after discharge. Additionally, pharmacists will conduct a post-discharge phone call with patients 30 days after the discharge to enquire about any DRPs, ED visits, or unscheduled readmissions, and their causes. The patients will be interviewed and asked specific questions pertaining to various aspects related to their health and medication. These questions include inquiries about possible side effects, any new symptoms experienced, history of outpatient or inpatient visits following their discharge, and any changes made to their current medications.

#### 2.4.2. Phase 2: Intervention group

Patients in the intervention group will receive multidisciplinary MR. Pharmaceutical patient care will be provided by six clinical pharmacists who have extensive experience in the MR process Additionally, they have a strong understanding of geriatric pharmacotherapy and, in this study, the clinical pharmacists are responsible for the entire MR service. Along with the nursing record of preadmission medications at the time of admission, pharmacists will obtain the BPMH by patient interview and “My Medication at a Glance” service provided by Health Insurance Review & Assessment Service (HIRA) before surgery. “My Medication at a Glance” is a government-run service that provides access to patient history of prescribed medications for the last 1 year with their consent. However, it only contains information on reimbursed drugs; therefore, we will interview patients to obtain accurate lists of medications that they are actually taking, including all prescribed medications, over-the-counter medications, and dietary supplements. Drug name, dose, frequency, indication, duration of therapy, brand, and route of administration will be noted. After acquiring the BPMH, the pharmacist documents it in electronic medical record and shares it with other healthcare professionals. Additionally, health professionals will measure frailty according to the Korean-translated version of the CFS.

During hospitalization, pharmacists will review the patient’s BPMH and compare it with the physician’s orders and in-hospital medication records. If any discrepancies are found, pharmacists will investigate the reason for the discrepancy in the EMR. If the reason is not documented in the EMR, doctors are consulted to determine whether the discrepancy is intentional or unintentional. If the discrepancy is intentional, doctors are encouraged to document the reasons in the EMR. If unintentional, the discrepancy will be mediated. If DRPs are identified, pharmacists and doctors will collaborate to resolve them.

Upon discharge, pharmacists will review discharge medications and compare them with the BPMH. Any discrepancies will be addressed, and pharmacists will provide counselling regarding discharge medications and medication changes. They will also provide patient-friendly updated written medication lists that include information on newly added, changed, and discontinued medications. If a patient is discharged on the weekend, pharmacist-led counselling will be conducted via telephone on the next weekday.

At the first outpatient visit after discharge, pharmacists will meet the patients and enquire about their health and medication issues, including DRPs, ED visits, and unscheduled readmission. Additionally, patients will be asked to complete a survey that includes questions about their satisfaction with the MR service and whether their understanding of their medications has improved. Similar to the control group, the pharmacists will contact the patients by phone 30 days after discharge to obtain information about their health and medication issues, including DRPs, ED visits, and unscheduled readmissions.

### 2.5. Outcomes Measurement

#### 2.5.1. Primary Outcome

The primary outcome is to evaluate the impact of MR on medication discrepancies between BPMH and medications at care transition in the intervention and control groups. In general, medication discrepancies are classified into three categories: intentional, undocumented intentional, and unintentional discrepancies. In this study, medication discrepancies are defined as unintentional discrepancies and undocumented intentional discrepancies. Medication discrepancies will be further classified into subcategories including medication omission; incorrect dose, route, or frequency; incomplete prescription; commission without indication; therapeutic duplication; and wrong medication [11].

#### 2.5.2. Secondary Outcome

The secondary endpoints are to (1) estimate the incidence rate of medication discrepancies at each transition and the discrepancy rate among the sources of information, (2) explore the impact of MR services on the medication appropriateness index (MAI) score [12], the rate of DRPs within 14 days and 30 days after discharge, 30 day mortality, ED visit rate, and readmission rate after discharge, (3) investigate the rate and acceptability of pharmacist interventions during hospitalization, and (4) explore patient satisfaction among the intervention group.

The DRP characteristics will be described using the Pharmaceutical Care Network Europe (PCNE) DRP classification [13]. The incidence rate of medication discrepancies will be calculated by dividing the number of patients with at least one discrepancy by the total number of patients in each group.

### 2.6. Sample Size

The sample size calculation is based on the previous literature that referred to the incidence of medication discrepancies. The percentage of patients with at least one medication discrepancy varied based on ranged from 14.1% to 81.9% based on population and illness [14,15,16,17]. In a prospective French study, after proactive MR, the proportion of patients with at least one medication discrepancy decreased significantly from 45.8% to 2.07% (*p* < 0.001) [18]. Another study assessed MR in patients scheduled to undergo surgery, and the medication discrepancy rate in the intervention group decreased from 40.2% to 12.9% compared to that in the non-intervention group [19]. Using the reference rate of medication discrepancies reported in a previous study, it was estimated that 40% of the patients could experience at least one medication discrepancy, and the intervention may reduce these discrepancies by 40%. Assuming an alpha error of 5%, statistical power of 80%, and a 10% patient loss, the calculated sample size was 216 for the intervention group and 108 for the control group.

### 2.7. Data Collection and Management

Data will be collected from all enrolled patients, including demographic information, such as age, sex, body mass index, height, and weight. Information regarding comorbidities, pre-and postoperative diagnoses, type of surgery, and any postoperative complications will also be collected. Additionally, medication lists at preadmission, admission, and discharge; length of hospital stay; and any readmission or ED visits and their reason within 30 days after the discharge will be documented. Table 1 presents the outcome variables for the evaluation period and the measuring instruments used.

### 2.8. Statistical Analysis

An intention-to-treat analysis will be conducted. The baseline characteristics will be summarized with descriptive statistics. Primary and secondary endpoints, except the rate and acceptability of pharmacist interventions during hospitalization, will be compared between the control and intervention groups. Categorical variables will be analyzed using the chi-square test or Fischer’s exact test, and continuous variables will be analyzed using the Student’s *t*-test or Mann–Whitney test. Data analysis will be performed after the data for the entire sample have been collected, using SAS version 9.4 (2017 SAS Institute, Cary, NC, USA) with a significance level of 0.05 and the limits of a confidence interval of 95%.

### 2.9. Patient and Public Involvement

Patients or the public were not involved in the study design or conduct of this study. However, patients in the intervention group will be asked to complete a survey assessing patient satisfaction with MR services.

### 2.10. Trial Status

This trial is proceeding with phase 2. The study was registered in CRIS (Registration Number: KCT0006813; Registered 7 December 2021).

## 3. Discussion

The MERITS trial aims to evaluate the effect of a multidisciplinary MR service on medication management and patient outcomes during transitional care in older patients undergoing thoracic and cardiovascular surgeries. To the best of our knowledge, the MERITS trial is the first prospective study to evaluate the impact of implementing MR services in the Republic of Korea.

This study protocol is prone to several limitations. First, it will be conducted at a single center and target a specific population, which may limit its generalizability to other settings. Second, the MR service will be implemented only during weekdays, which may result in negative outcomes because MR may be more necessary on weekends owing to limited staff and shifts. Third, due to the before–after study design, the study cannot be blinded, which increases the risk of bias. Therefore, this study will use strategies to reduce observer bias, such as providing minimal information to the medical staff in the control group.

Although these limitations are important, the results of this study may contribute to the development of multidisciplinary MR services in cardiothoracic wards and improve patient safety during care transitions.

## Figures and Tables

**Table 1:** Study procedure of MERITS study.

Time Point	Study Period				
	Enrolment and Allocation	Post-Allocation			
	Admission	Hospitalization	Discharge	First Outpatient Visit (about 2 Weeks after the Discharge)	30 Days after the Discharge
**Enrolment**					
Eligibility screen	X				
Informed consent	X				
Allocation	X				
**Intervention**					
Phase 1: Control group (December 2021~)				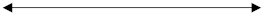	
Phase 2: Intervention group (After phase 1 ends~)		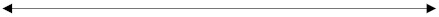			
**Assessments**					
Baseline characteristic	X	X			
Clinical Frailty Score		X			
Medication discrepancies	X	X	X		
Number of pharmacist interventions		X			
Medication appropriate index score			X		
Patient’s satisfaction				X	
Drug-related problems				X	X
Readmission				X	X
Emergency department visit				X	X
All-cause death				X	X

“X” and arrows show the data collection time points.

## Data Availability

Not applicable.
